# “Leaky Gut” as a Keystone of the Connection between Depression and Obstructive Sleep Apnea Syndrome? A Rationale and Study Design

**DOI:** 10.3390/metabo12020152

**Published:** 2022-02-06

**Authors:** Oliwia Gawlik-Kotelnicka, Aleksandra Margulska, Agata Gabryelska, Marcin Sochal, Piotr Białasiewicz, Dominik Strzelecki

**Affiliations:** 1 Department of Affective and Psychotic Disorders, Medical University of Lodz, 90-419 Lodz, Poland; dominik.strzelecki@umed.lodz.pl; 2 Central Teaching Hospital, Medical University of Lodz, 92-216 Lodz, Poland; aleksandra.margulska@gmail.com; 3 Department of Sleep Medicine and Metabolic Disorders, Medical University of Lodz, 90-419 Lodz, Poland; agata.gabryelska@umed.lodz.pl (A.G.); marcin.sochal@umed.lodz.pl (M.S.); piotr.bialasiewicz@umed.lodz.pl (P.B.)

**Keywords:** depression, obstructive sleep apnea, inflammation, oxidative stress, intestinal permeability

## Abstract

Obstructive sleep apnea (OSA) and depression are highly comorbid. Immune alterations, oxidative stress or microbiota dysfunction have been proposed as some mechanisms underlying this association. The aim of the proposed study is to assess the severity and profile of OSA and depressive symptoms in the context of serum microbiota metabolites, biomarkers of intestinal permeability, inflammation and oxidative stress in adult patients diagnosed with OSA syndrome. The study population consists of 200 subjects. An apnoea-hypopnoea index ≥ 5/hour is used for the diagnosis. Depressive symptoms are assessed with Beck Depression Inventory. Measured serum markers are: tumour necrosis factor–alpha and interleukin-6 for inflammation, total antioxidant capacity and malondialdehyde concentration for oxidative stress, zonulin, calprotectin, lipopolisaccharide-binding protein and intestinal fatty acids-binding protein for intestinal permeability. All of the above will be measured by enzyme-linked immunosorbent assay (ELISA). Associations between clinical symptoms profile and severity and the above markers levels will be tested. It would be valuable to seek for overlap indicators of depression and OSA to create this endophenotype possible biomarkers and form new prophylactic or therapeutic methods. The results may be useful to establish a subpopulation of patients sensitive to microbiota therapeutic interventions (probiotics, prebiotics, and microbiota transplantation).

## 1. Introduction

Metabolic disorders (metabolic syndrome with its complications and comorbidities) and depression are among the most disabling and common diseases worldwide. Moreover, they often coexist with each other thus increasing mortality risk [1]. Furthermore, a meta-analysis confirmed a reciprocal link between depression and obesity [2]. Another disorder that is strictly connected with both metabolic problems and depression is obstructive sleep apnoea (OSA). Its prevalence is increasing worldwide [3,4]. Metabolic disorders are in particular closely associated with OSA syndrome (OSAS) which is a chronic disorder characterized by intermittent hypoxia during sleep with re-oxygenation injury [5,6]. In fact, more than two thirds of people with OSA is at least overweight and weight loss helps reduce OSA severity or attenuate other symptoms (reviewed in [7]). Moreover, OSA is currently -regarded as an additional factor contributing to the pathogenesis of obesity-related comorbidities [8]. Multiple comorbid conditions such as metabolic complications (e.g., obesity, cardiovascular risk factors, ischemic heart disease and atrial fibrillation, impaired lipid levels, insulin resistance or type 2 diabetes mellitus and non-alcoholic fatty liver disease (NAFLD)), insomnia, depression (up to 35% [9]) or accelerated aging are particularly prevalent in individuals with OSA [3,5,10,11,12,13]. Among OSA patients there are those with extremely high prescription of antidepressants, anxiolytics, hypnotics and sedatives [3]. Importantly, comorbid mood symptoms developed by these patients may be misdiagnosed as a primary psychiatric condition thus it is important to search for OSA signals when examining patients with depression [14]. On the other hand, Reddy et al. found that prevalence of comorbid OSA in depression was 18% [15]. Additionally, it was established that atypical depression was a risk factor for OSAS in young adults [4]. In particular, patients with OSA and excessive daytime sleepiness (EDS) are more likely to have depressive symptoms as compared to controls [9,11,12,16,17]. Also, the reduced quality of life (QoL) is a strong predictor of psychiatric symptoms in OSAS patients [12]. In contrast, no correlation was observed between the overall severity of OSA and depression scores [9].

Few studies have investigated potential mechanisms that showed a relationship between OSA and depression. Several hypotheses have been proposed. First, OSAS is a chronic stressor that may influence the emotional state and QoL of patients suffering from it. However, the high co-occurrence rate suggests a possible partly pathophysiological overlap between depressive and sleep apnoea syndromes. The chronic low-grade inflammation (CLGI), oxidative stress, or microbiota dysfunction are all interplaying biological mechanisms that have been suggested [1,5,18,19,20].

Most civilization diseases, including depression and OSA, have been shown to be associated with CLGI. OSA symptoms lead to hypoxia and its severe pathological after-effect [5]. The cross-sectional epidemiological research found that oxygen saturation is a predictor of inflammation in the course of OSA [21]. Different studies have reported increased levels of inflammatory mediators in patients with OSA as compared to non-OSA subjects, however, their relationship with polysomnographic measures is controversial. On the contrary, they exhibit a positive correlation with the degree of adiposity [19,21]. On the other hand, emerging evidence suggests that chronic inflammation may mediate a part of depressive disorders cluster, especially atypical depression [22,23] and elevated plasma cytokines levels are common in major depressive disorder (MDD) [24,25].

There are also several studies which demonstrate that OxS (a state of imbalance between the pro-oxidative and anti-oxidative systems of cells and tissues) along with inflammation may be involved in the pathology of both OSA and depression. A pro-oxidant state in OSA may result from the recurrent hypoxia-reoxygenation cycles [5]. In a recent meta-analysis, blood superoxide dismutase (SOD) concentrations were shown to be significantly lower in OSA patients than those in the controls, which suggests an impaired antioxidant defence in OSA [20]. Total oxidant status or malondialdehyde (MDA) level (a proxy for lipid peroxidation) were reported to be higher, whereas total antioxidant capacity (TAC) lower in untreated MDD patients as compared to those of the controls [26,27]. Furthermore, antidepressant treatment proved to have a favourable impact on these parameters [26,28,29,30].

Recently, there has been a lot of interest in the role of gut microbiota changes in the pathophysiology of civilization diseases, including lifestyle behaviours and circadian rhythm disturbances, among others [31]. Changes in the intestinal microbiota has emerged to play a part in the occurrence of mood and anxiety disorders [32,33,34,35,36]. Additionally, the level of dysbiosis (a state of a disruption in the microbiota homeostasis) was shown to be associated with the severity of clinical depression [37,38]. Moreover, there is scientific data available on bidirectional connection of dysbiosis with hypoxia and its implication in the etiopathogenesis of OSA [39,40,41,42,43,44]. Animal model evidence strongly supports the idea that the impact of sleep fragmentation, intermittent hypoxia and intermittent hypercapnia on microbiota mediates disease states associated with OSA apnoea, including hypertension, atherosclerosis, and obesity [13,39].

Dysbiosis alters the permeability of the intestinal wall, and as a result products of the microbiota can induce systemic inflammation [45,46,47]. Importantly, recent studies have uncovered that psychological stress, including depression and suicidality, is associated with increased intestinal permeability [38,48,49,50,51,52]. Several biomarkers of this condition have been used, e.g., zonulin, calprotectin, lipopolysaccharide-binding protein (LBP) or intestinal-fatty acids binding protein (I-FABP) [53]. Generally, in patients suffering from a severe mental illness and chronic fatigue, a meta-analysis revealed increased levels of zonulin, lipopolysaccharide (LPS), antibodies against endotoxin, soluble CD14, LBP and, alpha-1-antitripsin as compared to controls [54]. However, little attention has been paid to the potential effects of intermittent hypoxia in OSA on the integrity and permeability of the intestinal barrier. In the cross-sectional study serum d-lactate was increased in non-obese males with OSA as compared to non-OSA subjects [55]. Furthermore, plasma I-FABP levels were significantly higher in patients with OSA than in controls and zonulin levels correlated negatively with the mean nocturnal oxygenation saturation [8].

Recently, short-chain fatty acids (SCFAs), such as acetate, propionate and butyrate, have been shown to have immunomodulatory properties and to play an important role in human health and disease [56]. Additionally, butyrate promotes intestinal barrier integrity depending on the presence of inflammation [57]. Fecal SCFAs are produced by intestinal microbiota from indigestible carbohydrates. Serum SCFAs (sSCFAs) are derived also from endogenous sources (metabolism of fat, carbohydrate, and amino acids) [56,58,59,60]. It was suggested that serum acetate is derived primarily from colonic fermentation, serum butyrate primarily from endogenous fatty acid metabolism, and serum propionate from both exogenous and endogenous sources [60]. Importantly, fecal acetate was likely to be positively associated with serum acetate [59,60]. Interestingly, sSCFAs levels were changed in some neuropsychiatric disorders, e.g., multiple sclerosis (MS) and Parkinson disease patients compared to controls [58,61,62,63]. Moreover, levels of sSCFAs were correlated with immune cell variety in MS subjects [64]. Furthermore, there was a significant reduction in serum propionate level in MS patients compared with controls [64]. Additionally, different sSCFAs levels correlated differently with the proinflammatory biomarkers [58]. Based on the above, it seems that the area of research on microbiota and inflammation in neuropsychiatric disorders can take advantage of the role of sSCFAs in intestinal-immune-brain connections [56].

## 2. Results

As a result of our investigation, we have constructed a study design presented below.

### 2.1. Aim of the Study

The aim of the study is the assessment of intestinal permeability, SCFAs levels, inflammation and OxS parameters depending on both the level of depressive symptoms and OSA severity in, both obese and non-obese, patients diagnosed with OSAS. The main confounder taken into account will be body mass index (BMI).

The study hypothesis is that comorbidity of depressive symptoms and OSAS is, at least partially, based on pathophysiological overlap involving inflammation, OxS, SCFAs levels and intestinal permeability changes.

The primary and secondary outcome measures are shown in Table 1.

### 2.2. Population

This study includes data from patients with presumptive diagnosis of OSAS assessed at the Department of Sleep Medicine and Metabolic Disorders of the Medical University of Lodz (in the period from January 2017 until now). All the patients undergo diagnostic polysomnography (PSG). The following inclusion criteria are applied in the study: age 18–70 years and body mass index (BMI) 20–45 kg/m^2^. Patients diagnosed with any chronic respiratory conditions (e.g., bronchial asthma, or chronic obstructive pulmonary disease) and any sleep disorders other than OSA (e.g., insomnia, delayed phase syndrome) are excluded from the study. Furthermore, the exclusion criteria are any infection (with or without antibiotic therapy), chronic inflammatory diseases (e.g., connective tissue diseases or inflammatory bowel diseases), diagnosis of cancer (active or recorded in a patient’s medical history), psychiatric disorders and shift work system, jet lag due to a flight within two weeks of the study or taking medications affecting sleep (e.g., benzodiazepines and melatonin).

The study population will include 200 subjects. Expert statistical advice has been sought when deciding on the number of participants in the project. As for sample size calculations, the authors have run several scenarios (Appendix A).

### 2.3. Measurements

Each participant completes a study questionnaire (SQ) to provide basic information concerning sociodemographic and health-related data (e.g., metabolic parameters as weight or body mass index (BMI), comorbidities, taken medications) and the 36-Item Short Form Health Survey (SF-36). An obstructive sleep apnoea-hypopnoea index (AHI) ≥ 5/hour is used for the diagnosis of OSAS (mild 5–14, moderate 15–29, severe ≥30). Depressive symptoms are assessed with the Beck Depression Inventory (BDI). Sleep-related factors severity were assessed with the Epworth Sleepiness Scale (ESS), the Pittsburgh Sleep Quality Index (PSQI), the Athens Insomnia Scale (AIS), the Insomnia Severity Index (ISI). The chronotype of each participant was determined.

Inflammation markers measured will be tumour necrosis factor–alpha (TNF-α) and interleukin-6 (IL-6). OxS blood level will be assessed as TAC and MDA concentration. Intestinal permeability will be assessed as surrogate biomarkers, i.e., zonulin, calprotectin, LPB and I-FABP concentration in blood serum. It has been shown that biomarkers from blood samples were associated with the cumbersome established tests of intestinal permeability throughout different cohorts [53]. Of SCFAs butyric, propionic and acetic acids level will be measured. All of the above will be measured in blood serum. Table 1 shows the summary of collected data.

### 2.4. Statistical Methods

We will try to assess the interplay between severity and the profile of depressive symptoms, severity of OSAS symptoms and levels of IL-6, TNF-alpha, MDA, TAC, zonulin, calprotectin, LBP and I-FABP. To verify the hypothesis that severity of depressive symptoms (as BDI score) may be related to severity of OSA (apnoea hypopnea index—AHI or ESS), the Spearman’s rank correlation coefficient will be used. All the patients will be grouped according to their BDI and AHI/ESS scores (into categories: none, low, medium high). Those subgroups as well as inflammation, intestinal permeability and OxS parameters (divided into categories: low, medium, high or within/over reference range) will be used for the Multiple Correspondence Analysis to establish a relationship between the abovementioned variables. The potential effect of the confounding factors will be tested in linear regression models. The model(s) will consist of age, sex, severity of OSA (AHI score/ESS), severity degree of depressiveness (BDI score), while MDA, TAC, IL-6, TNF-α, zonulin, calprotectin, LBP and I-FABP will serve as dependent variables. The Mann-Whitney U test and ANOVA Kruskal–Wallis tests will be performed in order to search for differences existing between the groups (with/without Dep, different severity levels of OSAS) as for OxS, inflammatory status and intestinal permeability parameters. The significance threshold for all the analyses will be set at *p* < 0.05. All the statistical analyses will be performed using Statistica 13.1 (StatSoft, Tulsa, OK, USA).

## 3. Discussion

**Figure 1:**
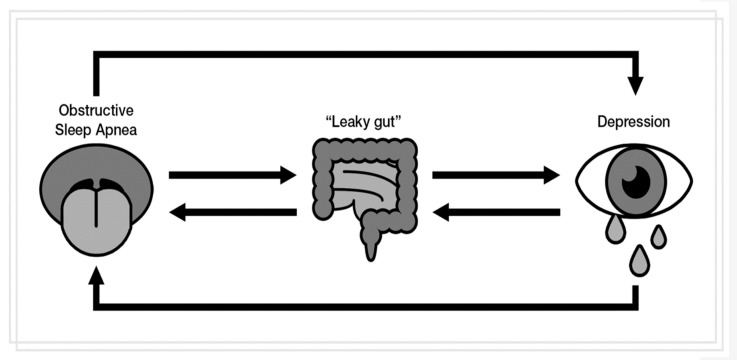
The simplified net of interconnections between OSA, depression and intestinal permeability.

There is more and more evidence which proves that an aberrant function of the gut microbiota and impaired intestinal barrier may be connected bidirectionally with CLGI [43,54] and OxS exacerbation in peripheral tissues and the brain [55]. Therefore this phenomenon may serve as a link between OSAS, depression and microbial metabolites and intestinal permeability. According to the ‘leaky gut hypothesis’, increased intestinal permeability may contribute to the relationship between civilization diseases and inflammation and, consequently, OxS via bacterial translocation across enterocytes [50]. Monocytes play an important role in inflammation and may be modulated by bacterial translocation. It was demonstrated that, as compared to the healthy controls, depressed patients showed an alteration in circulating monocytes and higher inflammatory state. At the same time, they showed increased LBP and I-FABP levels indicating more bacterial translocation and ‘leaky gut’ [56]. Furthermore, increased intestinal permeability in non-obese males with OSA (as compared to non-OSA subjects) showed significant associations with inflammatory mediators (serum IL-1β) [53], and zonulin levels in OSA patients correlated positively with some metabolic, inflammation and hepatic parameters [6]. Interestingly, OSAS children showed increased severity of inflammation and gut barrier damage-related strains as compared to healthy population [57]. Additionally, serum calprotectin was shown to correlate with clinical, biochemical (TNF-α and IL-6) and histological measures of intestinal inflammation [58]. Interestingly, supplemented propionate influenced positively T cells function and MS clinical picture [61]. Moreover, it was found that supplementation with probiotics (live microorganisms which, if consumed in adequate amounts, confer a health benefit on the host [59]) can decrease the levels of inflammatory markers in healthy and sick individuals [60,61] and restore, directly or indirectly, the oxidative balance [62,63]. Figure 1 shows the simplified net of interconnections between OSA, depression and ”leaky gut”.

Current treatments for both depression and OSAS (including its complications) remain suboptimal for many patients. Most of the studies revealed an improvement in OSA-related depressive and anxiety symptoms with continuous positive airway pressure (CPAP) therapy, however, the improvement is far from sufficient [65,66]. Additionally, lower CPAP adherence was shown to be an obstacle [67]. Specialized adjuvant therapies may be required in cases of residual or treatment-resistant mood symptoms. Elucidation of the above mechanisms linking OSAS, depressiveness, CLGI, SCFAs, OxS and intestinal permeability could generate new therapeutic targets or patient-specific strategies to combat both sleep apnoea and depressive disorders.

### Strengths and Limitations

The strength of our report is that, to our knowledge, such a thorough analysis of associations of sSCFA levels with intestinal permeability, inflammation and OxS serum biomarkers has not previously been performed in any of neuropsychiatic diseases. Specifically, the sSCFAs concentration in both depression and OSA patients is very poorly known. Concurrently, the immunomodulatory actions of sSCFAs in depression and OSA pathogenesis remain still uncertain.

Our study has also got several limitations. First of all, it should be emphasized that intestinal permeability ans sSCFAs levels are only a proxy indicators of intestinal dysbiosis and further studies in the field should incorporate stool microbiota examination. Furthermore, it is worth remembering that the BDI-II is widely used as an indicator of the severity of depression, but not as a diagnostic tool. This is the reason why we assess rather depressiveness, not a depression as a disease state. Nevertheless, numerous studies provide evidence for the BDI-II reliability and validity across different populations and cultural groups.

## 4. Materials and Methods

The Beck Depression Inventory II (BDI-II) is a world-wide 21-item self-rating scale for measuring intensity of depressive symptoms in the previous two weeks [68]. Analysis of BDI-II retrieved three factors: cognition, somatic complaints and affect. The study is being used the validated Polish translation of the scale [69].

Venous blood is collected by qualified nurses according to the reliable protocols of collection, transport, and storage of biological material. Twenty milliliters (20 mL) of whole arm vein blood is collected from each person. Samples of fasting blood will be collected from the subjects after overnight rest, in the morning, between 8:00 and 10:00 a.m. To obtain serum for future analysis (sSCFAs, TAC, MDA, Il-6, TNF-α, zonulin, calprotectin, LBP, I-FABP), blood is transferred to the sterile tubes without an anticoagulant (on the so-called clot) and left at room temperature (approximately 30–45 min) to form a clot. After centrifugation at 1000× *g* (2400 rpm) for 10 min, the serum (supernatant) is carefully separated from the clot into cryotubes. Approximately 3 mL of serum is obtained from each patient. Until further analysis is made, the blood samples is preserved in pyrogen/endotoxin-free collecting tubes (in 500 μL aliquots to avoid repeated freeze-thaw cycles) and stored frozen at −80 °C.

TNF-α, Il-6, TAC, MDA, LBP, zonulin, calprotection and I-FABP from blood serum will be measured by enzyme-linked immunosorbent assay (ELISA) kits. The procedures will be performed in compliance with the manufacturers’ instructions.

The measurement of SCFAs will be outsourced. An Sciex Triple TOF 6600+ equipped with an ExionLC AD series will be used. The LC flow rate will be 0.3 mL/min. The column used for the analysis will be a Kinetex Polar 2.6 µm (50 mm × 3 mm). The column temperature and auto sampler will be maintained at 20 °C and 4 °C, respectively. 1 µL will be used for the injection volume. Samples will be analysed using 10mM ammonium acetate in 80% methanol with 20% water (mobile phase A) and acetonitrile (mobile phase B). The isocratic elution will be 70% mobile phase A and 30% mobile phase B. The total run will be as 6 min. The Triple TOF 6600+ system will be equipped with an electrospray ionization (ESI) and Atmospheric-pressure chemical ionization (APCI) source operated in positive and negative-ion detection mode. Nitrogen gas will be used for nebulation, desolvation, and collision. The source parameters will be: gas temperature of 150 °C, a Source gas 1 and source gas 2 pressure of 50 psi and capillary voltage of −3500 V for negative polarity. Serum (200 µL) will be mixed with 200 µL acetonitrile and vortexed. Samples will be kept for 5 min on the ice to complete protein precipitation. After centrifugation for 10 min at 4 °C at 5000 rpm and filtered through a syringe filter 0.22 µm samples will be transferred to HPLC vials and analysed by the present LC-MS technique. A standard curve will be prepared by preparing serial dilutions of the standard mix.

## 5. Conclusions

Although evidence is limited, several studies suggest that treatment of OSA and associated cardiometabolic, neuropsychiatric or gastrointestinal disorders may be based on modulation of the microbiota through pro-, prebiotics, SCFAs, and faecal matter transplantation (FMT) [39,70]. However, to date, there are no clinical trials in this area.

Additionally, whilst microbiota interventions may provide benefits to some individuals, the target clinical sample for this intervention is not fully recognized. This trial will assess indirect markers of intestinal microbiota function, inflammatory status markers and OxS parameters as potential bioindicators of depressive/OSA subpopulation sensitive to add-on treatment with microbiota interventions. The trial, if successful, may establish an easy-to-use biomarkers for clinical practice. The idea is fresh and has a few innovative elements such as concurrent assessment of several clinical indicators, searching for common features of depression and OSAS in terms of polysomnographic, proxy intestinal microbiota, inflammatory and OxS parameters, as well as holistic insight into civilization diseases.

## Figures and Tables

**Table 1:** Primary (**in bold**) and secondary outcome measures.

Area of Interest		Material and Method	Potential Endophenotype Marker of OSA-Related Depression
General		SQ	Weight, BMI, comorbidities, medications
		SF-36	QoL
Clinical symptoms	Sleep-related	PSG	**AHI**
		ESS, PSQI, AIS, ISI	**Daily sleepiness**, sleep quality, insomnia, chronotype
	psychological	BDI	**Depressiveness**
Inflammation		Blood serum, ELISA	TNF-α, Il-6
Oxidative stress		Blood serum, ELISA	TAC, MDA
Intestinal permeability		Blood serum, ELISA	**Zonulin, LBP, I-FABP, Calp**
Microbiota function		Blood serum,	**SCFAs**

Abbreviations: AHI: apnoea-hypopnea index; AIS: Athens Insomnia Scale; BDI: Beck Depression Inventory; BMI: body mass index; Calp: calprotectin; ELISA: enzyme-linked immunosorbent assay; ESS: Epsworth Sleepiness Scale; I-FABP: intestinal fatty acids-binding protein; Il-6: interleukin-6; ISI: Insomnia Severity Index; LBP: lipopolisaccharide-binding protein; MDA: malonyldialdehyde; PSG: polysomnography; PSQI: Pittsburgh Sleep Quality Index; QoL: quality of life; SCFAs: short-chain fatty-acids; SF-36: 36-Item Short Form Health Survey; SQ: study questionnaire; TAC: total antioxidant capacity; TNF-α: tumour necrosis factor–alpha.

## Data Availability

Not applicable.

## Appendix A. Sample Size Analyses Details

### Appendix A.1. Correlation

**Depressiveness and EDS**: Using online calculator (https://sample-size.net/correlation-sample-size/ - accessed on 15 December 2021) we assessed that the total sample size required to determine whether a correlation coefficient differs from zero is N = 194 (the expected correlation coefficient r = 0.2, power–80%, α = 0.05). More detailed approach indicates that this number may be smaller. If we were to detect a moderate correlation *r* (*r* = 0.4) of observations, then using a two-sided test, 5% significance level test (*α* = 0.05) with power 80% power (β = 0.2), the required sample size would be approximate *n* = 47. S. Ishman et al. [9] found a significant correlation between BDI and Epworth Sleepiness Scale (ESS) scores (r = 0.342, *p* = 0.012). Hence to find a correlation between depressiveness and severity of OSAS we may need 66 subjects (r = 0.342, power 80%, α = 0.05). Daabis et al. [12] found even greater correlation between depressiveness (HADS-D score) and ESS r = 0.8 (*p* = 0.002).

**TAC**. In the study by Baek et al. [30] the correlation between TAC and BDI was r = −0.383. There are studies showing even greater correlation between severity of depression with MADRS scale and TAC: r = −0.553 (study by Cumurcu et al. [26]). Therefore we assume that to find statistically significant correlation between BDI or OSAS score and TAC or MDA the group of 51 patients may prove sufficient (the expected correlation coefficient r = 0.383, power—80%, α = 0.05).

**I-FABP**. In the study by Ohlsson et al. [52] the correlation between I-FABP and depressiveness level (by MADRS and SUAS score) was significant and the correlation coefficient was from r = 0.25 up to 0.60. Therefore we assume that to find statistically significant correlation between I-FABP and BDI score the group of 170 patients may prove sufficient (the expected correlation coefficient r = 0.30, power—80%, α = 0.05).

### Appendix A.2. Comparing Two or More Groups

Studies have shown that distributions of oxidative stress [71], intestinal permeability [72] and inflammatory parameters [73,74] may not be normal. M. Burg et al. demonstrated that distribution of BDI scores are not normal [75]. Vasas et al. reported that ESS distribution tends to be right-skewed [76]. Some researchers suggested data for cytokines were transformed into normal distribution using the natural logarithms before statistical analysis [77].

**MDA**. In the study by Taene et al. MDA has mean of 6.16 μmol/L in depressed subjects and 3.32 μmol/L in non-depressed subjects and a standard deviation of 2 (the difference between groups was statistically significant). Effect size in such case may be relatively high-Cohen d was 1.4. Difference in MDA of 0.8 or higher seems clinically relevant (then Cohen’s D has value of 0.4).

Using online tool (stat.ubc.ca) we calculated a sample size (assuming that both groups taking part in the study are the same size). Each sample size for this study should be around 99 (M1 = 3.8, M2 = 3.0, SD = 2, alpha = 0.05, power = 80%).

We verified that numbers using Statistica 13. If the statistical values are as follows M1 = 3.8, M2 = 3.0, SD = 2, n1 = 99, n2 = 99, power goal = 0.80, then we get statistical significance of 0.005.

To sum up, to find the difference between groups as for MDA levels, the required number of subjects is 100 per group.

**TAC**. In the study by Taene et al. [27] TAC has mean of 2.31 μmol/L in depressed subjects and 3.08 μmol/L in non-depressed subjects and a standard deviation of 0.54 (the difference between groups was statistically significant). Effect size in such case was relatively high-Cohen d was 1.4. Difference in TAC of 0.22 or higher seems clinically relevant (then Cohen’s D has value of 0.4).

In the study by Cumurcu [26] et al. TAC has mean of 1.10 μmol/L in depressed subjects and 1.31 μmol/L in non-depressed subjects and a standard deviation of 0.12 (the difference between groups was statistically significant). Effect size in such case may be relatively high-Cohen d was 1.75. Difference in TAC of 0.05 or higher seems clinically relevant (then Cohen’s D has value of 0.4).

Using online tool (stat.ubc.ca) we calculated a sample size (assuming that both groups taking part in the study are the same size). Each sample size for this study should be around 91 (M1 = 1.15, M2 = 1.20, SD = 0.12, alpha = 0.05, power = 80%).

We verified that numbers using Statistica 13. If the statistical values are as follows M1 = 1.15, M2 = 1.2, SD = 0.12, n1 = 91, n2 = 91, power goal =0.80, then we get statistical significance of 0.006.

To sum up, to find the difference between groups as for TAC levels, the required number of subjects is 91 per group.

**IL-6**. In the study by Janelidze et al. [77] IL-6 has mean of 3.8 pg/mL in depressed subjects and 3.5 pg/mL in non-depressed subjects and a standard deviation of 2.5 (the difference between groups was not statistically significant). Difference in IL-6 of 1 or higher seems clinically relevant (then Cohen’s D has value of 0.4).

Using online tool (stat.ubc.ca) we calculated a sample size (assuming that both groups taking part in the study are the same size). Each sample size for this study should be around 99 (M1 = 3.0, M2 = 4.0, SD = 2.5, alpha = 0.05, power = 80%).

We verified that numbers using Statistica 13. If the statistical values are as follows M1 = 3.0, M2 = 4.0, SD = 2.5, n1 = 99, n2 = 99, power goal =0.80, then we get statistical significance of 0.005.

To sum up, to find the difference between groups as for IL-6 levels, the required number of subjects is 99 per group.

**TNFα**. In the study by Yao et al. [78] TNFα has mean of 7 fmol/L in depressed subjects and 3.2 fmol/L in non-depressed subjects and a standard deviation of 3.2 (the difference between groups was statistically significant). Difference in TNFα of 1.28 or higher seems clinically relevant (then Cohen’s D has value of 0.4).

Using online tool (stat.ubc.ca) we calculated a sample size (assuming that both groups taking part in the study are the same size). Each sample size for this study should be around 98 (M1 = 4.0, M2 = 5.28, SD = 3.2, alpha = 0.05, power = 80%).

We verified that numbers using Statistica 13. If the statistical values are as follows M1 = 4.0, M2 = 5.28, SD = 3.2, n1 = 99, n2 = 99, power goal =0.80, then we get statistical significance of 0.006.

To sum up, to find the difference between groups as for TNFα levels, the required number of subjects is 98 per group.

**Zonulin**. In the study by Maget et al. [79] zonulin has mean of 63.7 ng/mL in depressed subjects and 51.6 ng/mL in euthymic subjects and a standard deviation of 31.7 (the difference between groups was not statistically significant). Difference in zonulin of 12.7 or higher seems clinically relevant (then Cohen’s D has value of 0.4).

Using online tool (stat.ubc.ca) we calculated a sample size (assuming that both groups taking part in the study are the same size). Each sample size for this study should be around 98 (M1 = 63.7, M2 = 51, SD = 31.7, alpha = 0.05, power = 80%).

We verified that numbers using Statistica 13. If the statistical values are as follows M1 = 63.7, M2 = 51, SD = 31.7, n1 = 98, n2 = 98, power goal =0.80, then we get statistical significance of 0.02.

To sum up, to find the difference between groups as for zonulin levels, the required number of subjects is 98 per group.

### Appendix A.3. Multiple Correspondence Analysis

As it is an exploratory technique there are no strict guidelines for sample size. We assume that our study population will exceed 200 subjects.

### Appendix A.4. Regression

Jenkins et al. [80] demonstrated that for biological and medical purposes the minimum number of observation required to successfully perform regression analysis might be as small as 25.
